# Characterization of atrial and ventricular remodeling in an improved minimally invasive mouse model of transverse aortic constriction

**DOI:** 10.20517/jca.2023.18

**Published:** 2023-07-10

**Authors:** Jose Alberto Navarro-Garcia, Satadru K. Lahiri, Yuriana Aguilar-Sanchez, Anilkumar K. Reddy, Xander H. T. Wehrens

**Affiliations:** 1 Cardiovascular Research Institute, Baylor College of Medicine, Houston, TX 77030, USA; 2 Department of Integrative Physiology, Baylor College of Medicine, Houston, TX 77030, USA; 3 Department of Medicine/DeBakey Heart Center, Baylor College of Medicine, Houston, TX 77030, USA; 4 Department of Neuroscience, Baylor College of Medicine, Houston, TX 77030, USA; 5 Department of Pediatrics, Baylor College of Medicine, Houston, TX 77030, USA; 6 Center for Space Medicine, Baylor College of Medicine, Houston, TX 77030, USA

**Keywords:** Atrial remodeling, atrial fibrillation, heart failure, mouse model, transverse aortic constriction

## Abstract

**Introduction:**

Heart failure (HF) is the leading cause of death worldwide. Most large and small animal disease models of HF are based on surgical procedures. A common surgical technique to induce HF is transverse aortic constriction (TAC), which induces pressure overload. The conventional TAC (cTAC) procedure is a highly invasive surgery that is associated with severe inflammation and excessive perioperative deaths.

**Aim:**

To establish an improved, minimally invasive TAC (mTAC) procedure that does not require thoracotomy.

**Methods and results:**

Following anesthesia, mice were intubated, and a small incision was made at the neck and chest. After cutting the sternum about 4 mm, the aortic arch was approached without opening the pleural cavity. A suture was placed between the brachiocephalic artery and the left common carotid artery. This model was associated with low perioperative mortality and a highly reproducible constriction evidenced by an increased right-to-left carotid blood flow velocity ratio in mTAC mice (5.9 ± 0.2) *vs*. sham controls (1.2 ± 0.1; *P* < 0.001). mTAC mice exhibited progressive cardiac remodeling during the 8 weeks post-TAC, resulting in reduced left ventricular (LV) contractility, increased LV end-systolic diameter, left atrial enlargement and diastolic dysfunction, and an increased heart weight to tibia length ratio (mTAC: 15.0 ± 0.8 *vs*. sham: 10.1 ± 0.6; *P* < 0.01).

**Conclusion:**

Our data show that the mTAC procedure yields a highly reproducible phenotype consisting of LV contractile dysfunction and enlargement, combined with left atrial enlargement and diastolic dysfunction.

**Potential impact of the findings:**

This model may be used to test the molecular mechanisms underlying atrial remodeling associated with HF development or to evaluate therapeutic strategies to treat these conditions.

## Graphical Abstract

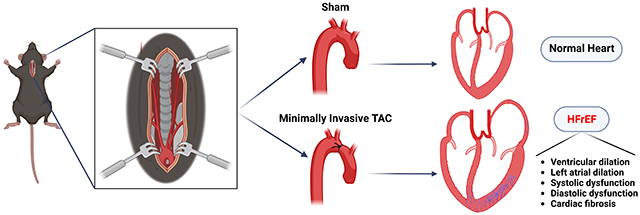

## INTRODUCTION

Atrial fibrillation (AF) and heart failure (HF) are both very common, progressive diseases that often co-exist, predispose to each other, and share risk factors. Both diseases share common structural alterations (e.g., fibrosis, inflammation, chamber dilatation) that point to overlapping underlying molecular mechanisms. HF is a disease characterized by the inability of the heart to supply the peripheral tissue with enough blood and oxygen for its metabolic requirements due to cardiac dysfunction^[1]^. HF with a preserved ejection fraction (HFpEF) develops when the left ventricle becomes more rigid, and relaxation is impaired; this is most common in older people and women^[2]^. On the other hand, in HF with a reduced EF (HFrEF), systolic HF develops with reduced left ventricular (LV) contractility. HFrEF is often caused by high blood pressure and coronary artery disease. These risk factors are also known to cause AF, an irregular and often very rapid heart rhythm abnormality that can reduce cardiac output and promote strokes^[3]^. A substrate for AF often develops in the atria when the ventricles undergo adverse remodeling due to the development of HF. New and more efficient treatments are needed and require an improved understanding of the disease mechanisms. Reproducible animal models of HF are critical for both the knowledge of disease pathophysiology and the development of new treatment modalities.

A commonly used small animal model for HF is pressure overload induced by transverse aortic constriction (TAC)^[4,5]^. This model, first described by Rockman *et al.* in 1991, consists of a permanent constriction at the aortic arch that limits blood flow across the aortic valve, thus subjecting the left ventricle (LV) to increased pressure load^[6]^. More specifically, constriction is created between the brachiocephalic (innominate) artery and the left common carotid artery with either a surgical suture or a small rubber ring (O-ring-induced TAC)^[7]^. It is well established that TAC leads to ventricular remodeling that initially promotes compensated hypertrophy but eventually results in adverse cardiac remodeling, fibrosis, and systolic/diastolic dysfunction, leading to HF progression^[8]^. In addition, it has been shown that pressure overload can cause atrial remodeling and fibrosis^[9]^.

Researchers commonly use the conventional surgical model of TAC (cTAC), which involves the opening of the thorax and pleural cavity^[6]^. We recently reported that thoracotomy *per se* induces cardiac abnormalities such as atrial arrhythmias, even in the absence of any overt cardiac damage^[3]^. To minimize the potential adverse effects of the surgical procedure on cardiac function, we characterized a minimally invasive TAC (mTAC) model in which the pleura is kept intact during the surgical procedure. This model permits the study of the effects of pressure overload on the heart, devoid of the robust inflammation process in the thorax that accompanies the cTAC surgery. The mTAC model has been previously described^[10]^ in terms of the surgical procedure as the model of ventricular pressure overload^[11]^; however, the pathophysiological consequences in the different cardiac chambers have not been described. Here, we report the first in-depth analysis of chamber-specific remodeling and functional consequences of the non-invasive pressure overload procedure in the mTAC mouse model.

## METHODS

### Animal studies.

All studies were performed according to protocols approved by the Institutional Animal Care and Use Committee (IACUC) of Baylor College of Medicine, conforming to the Guide for the Care and Use of Laboratory Animals published by the U.S. National Institutes of Health (NIH Publication No. 85-23, revised 1996). All imaging and histological studies were performed by investigators blinded to the surgical procedure performed in mice at Baylor College of Medicine.

### Minimally invasive transverse aortic constriction (mTAC).

**Figure 1.**
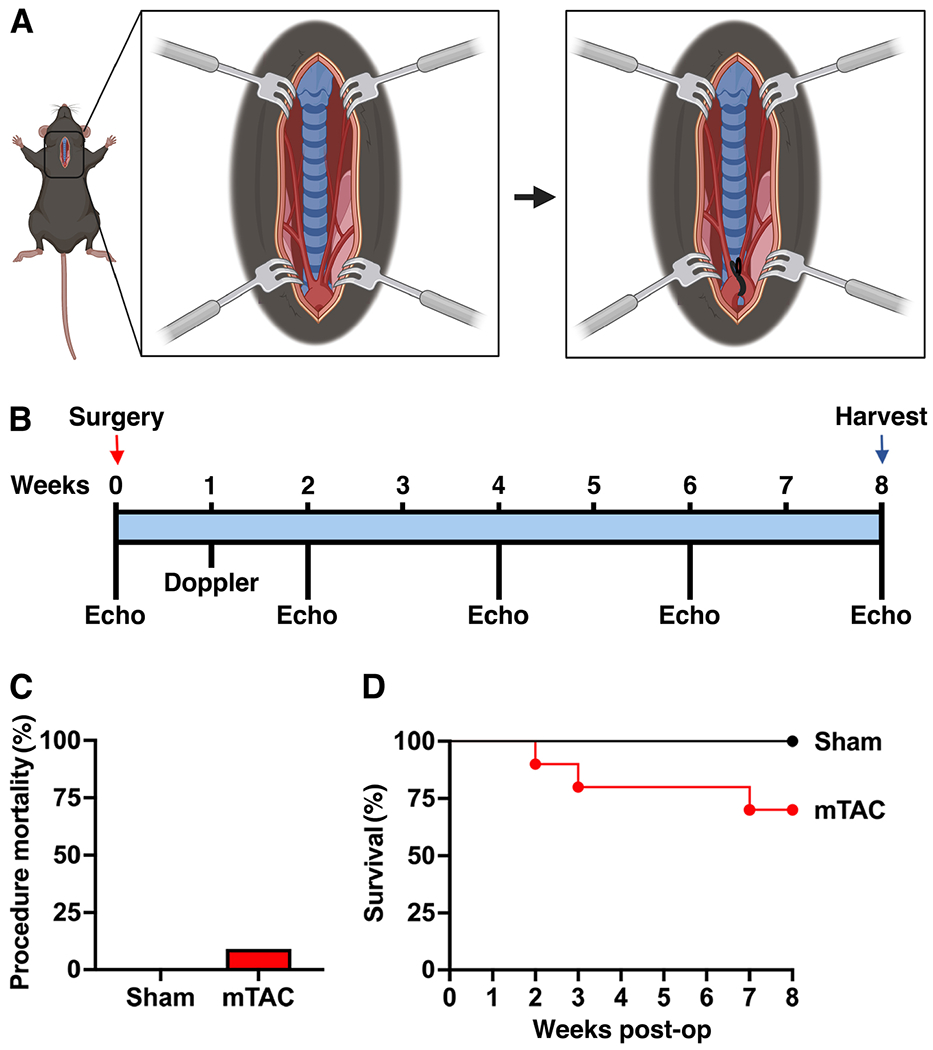
Overview of minimally invasive transverse aortic constriction (mTAC) model. (A) A schematic illustration of the incision exposing the aortic arch and ligation site. (B) Flow chart of the mTAC surgical model with time points for echocardiography (echo). (C) Procedure-derived mortality during mTAC surgery in sham (*n* = 6) and mTAC mice (*n* = 11). (D) Survival Kaplan-Meier curve for sham (black line, *n* = 6) and mTAC (red line, *n* = 10) mice during the 8-week postoperative (post-op) period. The student’s *t*-test was used to compare procedure-derived mortality and the Mantel-Cox test was used for survival Kaplan-Meier curve comparison.

Adult 12- to 14-week-old C57BL/6J male mice were randomized to sham and mTAC groups. Slow-release (SR) Buprenorphine (1 mg/kg/72 h) and SR Meloxicam (2 mg/kg/48 h) were subcutaneously injected 1 h prior to surgery. Mice were placed in the supine position on a heating pad during surgery to maintain their body temperature at 37.0 ± 0.5 °C. Following induction of anesthesia in a chamber with 3% v/v isoflurane in 100% oxygen, mice were intubated and ventilated at a tidal volume of 150 μL and respiratory rate of 175 breaths/minute. Anesthesia was maintained with inhaled isoflurane (2% v/v, isoflurane/oxygen). A small incision of around 2 cm was made at the midline position of the neck and chest. The thyroid gland was pulled towards the head and muscle layers on the trachea were separated at the midline to both sides with the help of curved forceps. Muscles surrounding the trachea were separated from behind the sternum using blunted scissors. The sternum was cut about 4 mm, from the top down to the second rib, and retractors were used to keep the incision open. The aortic arch and both carotids were clearly visible at the lower part of the incision [Figure 1A]. Using curved blinded forceps, a tunnel was created under the aortic arch and between the first (brachiocephalic artery) and second (left common carotid artery) branches of the aorta. A non-absorbable 6-0 monofilament silk suture was crossed through the tunnel to encircle the aortic arch. A blunt 27-gauge needle was placed over the arch and used as a spacer. Then, the suture was fixed in place with a double knot and the spacer was gently removed [Figure 1A]. The chest was closed by crossing a 6-0 monofilament absorbable suture through the first intercostal space from both sides of the cut. The skin over the chest was closed using a 6-0 monofilament non-absorbable suture in a non-continuous suture pattern. Skin glue was used to ensure that the incision remained closed. The same procedure was performed in sham mice, except that no ligation was performed on the aortic arch.

### Doppler measurements.

A high-frequency pulsed Doppler ultrasound system (DVFS, Indus Instruments, Webster, TX) was used to measure blood flow velocity in the carotid arteries after surgery. Doppler studies were performed one week after surgery in both carotid arteries for sham and mTAC mice [Figure 1B]. During the imaging study, mice were placed on a heating board (Rodent Surgical Monitor (RSM)+, Indus Instruments, Webster, TX) in a supine position and anesthetized with isoflurane (1.5% v/v, isoflurane/oxygen). The RSM+ board includes electrocardiographic electrodes and a heating pad. Body hair was removed for improved ultrasound coupling between the probe and the skin, and ultrasound transmission gel was applied to the skin. A 20 MHz Doppler probe was placed on both the right and left sides of the neck at about 45° angle to measure blood flow velocity in both carotid arteries. Doppler range gate depth was adjusted to 2-3 mm to obtain the optimal flow velocity signals. The probe used in this study was custom-built in Dr. Reddy’s laboratory, as described^[12,13]^. The ratio between right and left carotid flow (RC/LC) was used as an indicator of the severity of the constriction.

### Echocardiography measurements.

Baseline echocardiography was performed 3 days prior to sham/mTAC procedures and at 2-, 4-, 6- and 8-weeks post-surgery [Figure 1B]. Mice were anesthetized with isoflurane (1.5%-2% v/v, isoflurane/oxygen). After the chest hair was removed, mice were placed on a heated platform to maintain the body temperature at 37.0 ± 0.5 °C. Echocardiography was performed using Visual Sonics Vevo 2100 with a 30 MHz frequency probe (FujiFilm VisualSonics Inc., Toronto, ON, Canada). Short-axis images of the heart in both B- and M- modes were recorded to assess systolic function. Long-axis B-mode images were captured to determine the left atrial area. Long axis and 4-chamber color Doppler/pulsed wave were used to assess pulmonary artery flow and mitral valve early (E) and after (A) wave. Long-axis imaging of the upper chest was used to image the aortic arch. All captured images were analyzed using Vevo LAB software.

### Histology.

Mice were euthanized following induction of anesthesia with isoflurane (3% v/v, isoflurane/oxygen), followed by cervical dislocation and bilateral opening of the thorax. Mouse hearts were fixed with 10% buffered formalin and dehydrated in an increasing percentage of ethanol series. Both xylene and paraffin washes were used to paraffinize the samples. Paraffinized hearts were sectioned in 7 μm sections and placed on slides. Tissue sections were further deparaffinized with a decreased percentage of ethanol series and were stained with Masson’s Trichrome stain (Thermo Fisher Scientific, Waltham, MA, USA) to label fibrosis. Stained heart sections were imaged using a light microscope. The fibrotic area (blue-positive) was quantified using ImageJ^[14]^.

### RNA isolation and quantitative PCR (qPCR).

Total RNA was isolated from the whole heart of sham and mTAC mice. Hearts were snap frozen in liquid nitrogen and powdered. A small portion of the powder was used for RNA isolation and 400 μL of TRIzol per each sample. The Direct-zol RNA MiniPrep (Zymo Research, Irvine, CA) was used to isolate total RNA and RNA concentrations were measured using a nanodrop. RNA was stored at −80 °C. To generate cDNA, reverse transcription was carried out using the iScript Reverse Transcription Supermix (BioRad, Hercules, CA) with 500 ng of RNA per sample. Quantitative polymerase chain reaction (qPCR) was performed for NPPA and NPPB. A total of 20 μL reaction solution was used per well: 10 μL of SYBR Green Master Mix (Thermo Fisher Scientific), 1 μL of 4 × Yellow Sample Buffer (Thermo Fisher Scientific), 1 μL of 10 μM forward primer, 1 μL of 10 μM reverse primer, 2 μL of cDNA (prepped as described above), and 5 μL of distilled water. Fold changes in gene expression were calculated using the delta-delta Ct method. Gene expression fold changes (relative to *L7*) in mTAC hearts were then normalized to those in sham by dividing by the average sham fold change.

### Statistical analysis.

All data are expressed as mean ± SEM. GraphPad Prism 9 (GraphPad Software Inc., San Diego, CA, USA) was used to perform an unpaired Student *t*-test or ANOVA after performing the D’Agostino-Pearson normality test for normal data distribution. A one-way ANOVA or Kruskal-Wallis test was used for multiple group comparisons based on the data distribution. *P* < 0.05 was considered statistically significant.

## RESULTS

A total of 17 male 12- 14-week-old mice were used for this study. Mice were randomized into the sham (*n* = 6) and minimally invasive transverse aortic constriction (mTAC) groups (*n* = 11). Following intubation, mice underwent a surgical procedure that did not require a thoracotomy and opening of the pleural cavity, as described in the methods section. Intraoperative mortality (defined as any mortality during the first 24 h after the start of the surgery) was 0% and 9% for sham and mTAC, respectively [Figure 1C]. Intraoperative mortality was mainly due to bleeding from the aortic arch during the ligation process. Postoperative mortality for the mTAC group was found to be higher during the first week after the surgery process. The survival 8 weeks after surgery was 100% and 70% for sham and mTAC, respectively [Figure 1D]. Interestingly, mTAC did not significantly increase the mortality rate compared to sham-operated mice during the postoperative follow-up period of 8 weeks (*P* = 0.156).

**Figure 2.**
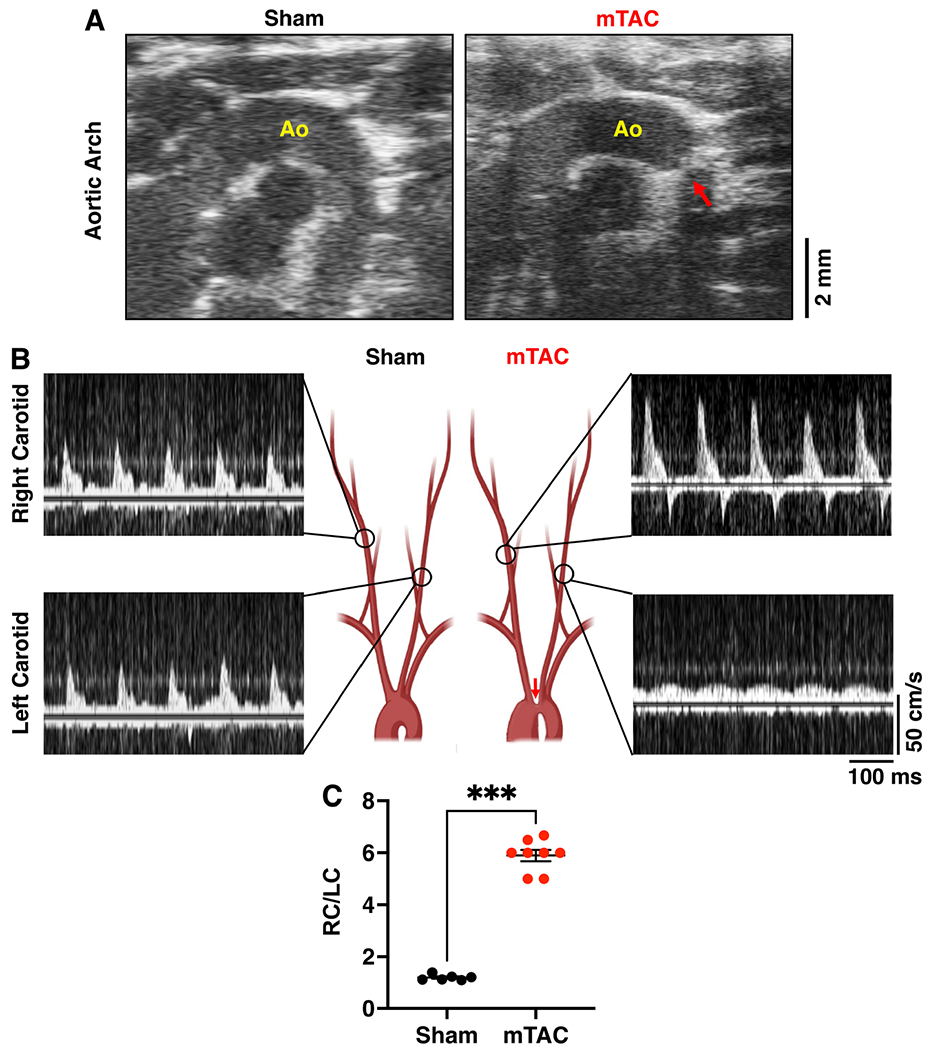
Effectiveness of aortic constriction in the mTAC model. (A) Representative echocardiography images of the aortic arch in sham (left panel) and mTAC (right panel) mice, showing the constriction (red arrow) of the aorta (Ao). (B) Schematic illustration of the aortic arch and representative recording of blood flow in right carotid (RC; upper panels) and left carotid (LC; bottom panels) from sham (left) and mTAC (right) mice. (C) Quantification of RC/LC ratios in sham (*n* = 6) *versus* mTAC (*n* = 8) mice. Data shown mean ± SEM. ***P < 0.001 using student’s *t*-test.

The constriction was placed between the brachiocephalic artery and the left common carotid artery and was validated by echocardiography studies [Figure 2A]. The presence of a constriction was clearly visible in all (10 out of 10) surviving mTAC mice. Additionally, high-frequency pulsed Doppler ultrasound studies were performed one week after surgery. Blood flow velocity was measured in both the right carotid (RC) and left carotid (LC) arteries. Representative recordings of the blood flow velocity from both carotid arteries in sham and mTAC mice are shown in Figure 2B. The RC blood flow velocity was much higher in mTAC mice, while LC blood flow velocity was decreased compared to sham mice. The ratio between right-to-left carotid flow velocity (RC/LC) was significantly increased 5.9-fold in mTAC mice *versus* the sham group [Figure 2C, *P* < 0.001], consistent with a moderate-to-severe rate of aortic arch constriction.

**Figure 3.**
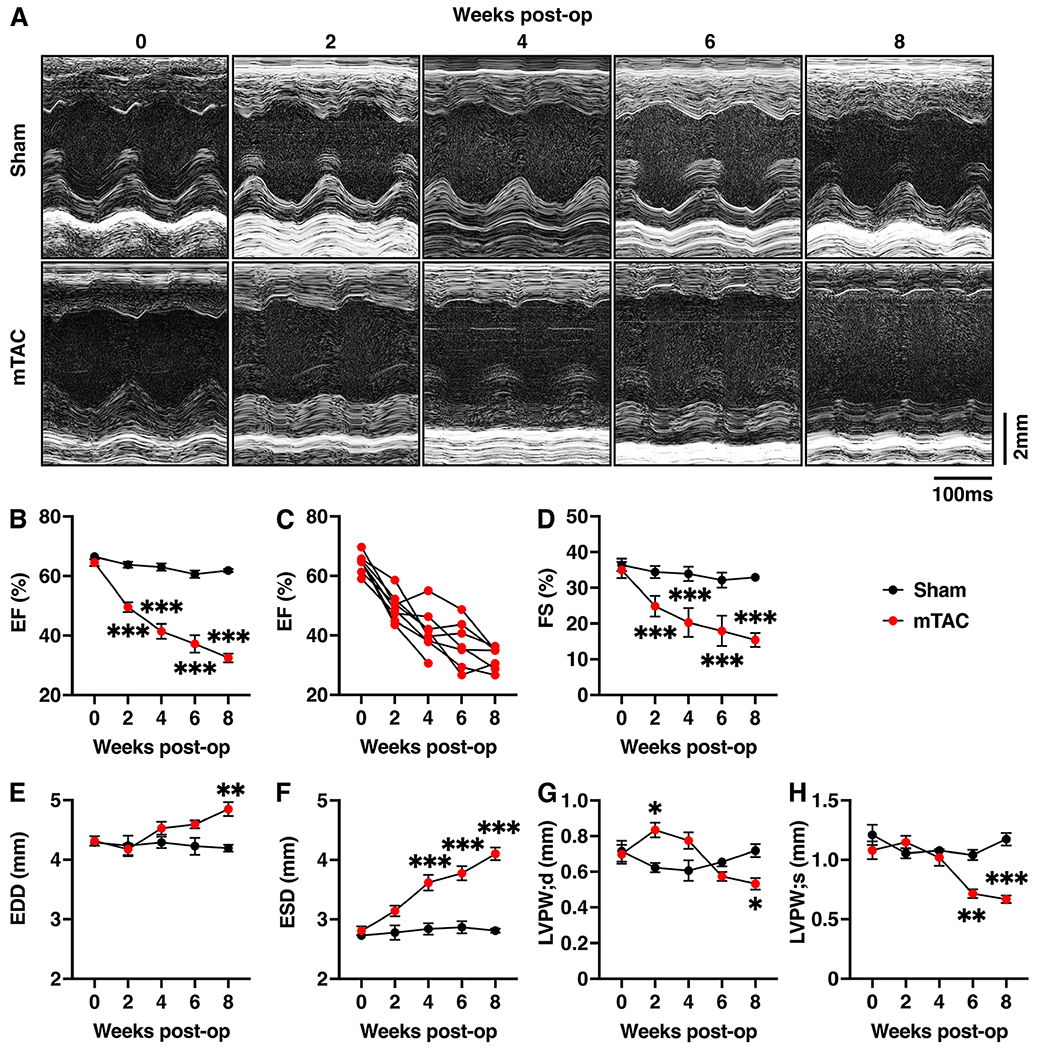
Heart failure with reduced ejection fraction induced by mTAC. (A) Representative M-mode echocardiography tracings from the left ventricle of sham (upper panels) and mTAC mice (bottom panels) at baseline and 2, 4, 6, and 8 weeks after surgery. (B) Average ejection fraction (EF) at the different time points in sham (black plots, *n* = 6) and mTAC (red plots, *n* = 8) mice. (C) Individual EF changes in mTAC mice at different time points. (D-G) Average of (D) fractional shortening (FS), (E) end-diastolic diameter (EDD), (F) end-systolic diameter (ESD), (G) left ventricular posterior wall at diastole (LVPW;d), and (H) LVPW in systole (LVPW;s), at the different timepoints in sham and mTAC mice. Data shown mean ± SEM. *P < 0.05, **P < 0.01 and ***P < 0.001, using 2-way ANOVA with Bonferroni’s multiple-comparision correction.

Echocardiography measurements were performed at baseline and 2-, 4-, 6-, and 8-weeks after surgery. Representative M-mode images for each time point are shown in Figure 3A. Data for both groups were similar at baseline [Figure 3 and Table 1]. Progressive systolic contractile dysfunction was observed in mTAC mice after the surgery. The ejection fraction (EF) was significantly decreased in mTAC mice compared to sham mice starting from the second week after surgery [Figures 3B, *P* < 0.001]. Further analysis revealed that each mTAC mouse exhibited a lower EF over time [Figure 3C]. Similarly, fractional shortening (FS) was also significantly reduced [Figure 3D, *P* < 0.001]. The cardiac output (CO) was also decreased in mTAC mice compared with sham mice [Table 1, *P* < 0.01].

End-diastolic diameter (EDD) and end-systolic diameter (ESD) were significantly increased in mTAC mice compared to sham mice. The EDD progressively increased and became statistically significant at 8 weeks post-surgery [Figure 3E, *P* < 0.05]. The ESD was significantly higher in mTAC mice starting from the fourth week after surgery [Figure 3F, *P* < 0.001]. Stroke volume (SV) was significantly lower in mTAC mice compared to sham mice starting from the second week after surgery [Table 1, *P* < 0.001]. Finally, the left ventricular posterior wall diastolic diameter (LVPW;d) was increased only at 2 weeks after surgery [Figure 3G], consistent with the typical hypertrophic response, which is usually followed by progression to a decompensated dilated cardiomyopathy^[15]^. Subsequently, the LVPW;d and LVPW in systole (LVPW;s) were significantly lower in mTAC mice starting at 8- and 6-weeks post-surgery, respectively [Figure 3G and H], indicative of LV dilatation associated with HF development.

**Figure 4.**
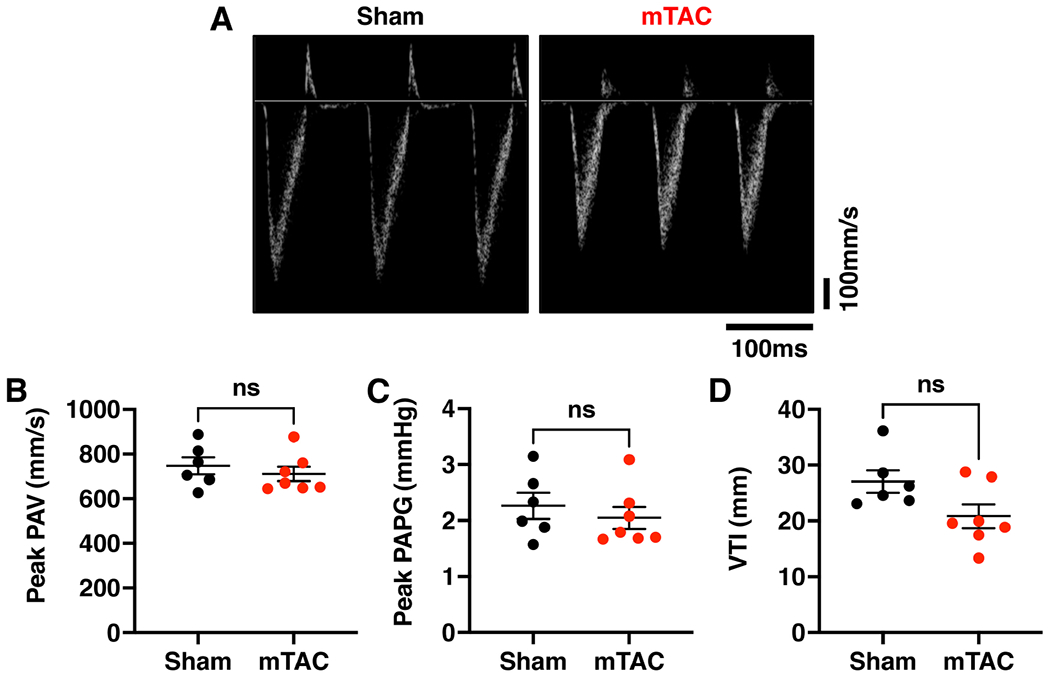
Preserved right ventricular function in mTAC mice. (A) Representative Doppler traces of pulmonary artery flow in sham (left panel) and mTAC mice (right panel). (B-D) Average values of (B) pulmonary vein artery velocity (PAV), (C) peak gradient (PAPG), and (D) velocity time integral (VTI) in sham (black dots, *n* = 6) and mTAC mice (red dots, *n* = 7). Data shown mean ± SEM. Student’s *t*-test was used to compare sham and mTAC groups.

To assess right ventricular (RV) remodeling post mTAC surgery, we used color Doppler echocardiography to measure blood flow through the pulmonary artery. The pulsed wave Doppler ultrasound was placed over the pulmonary artery in the parasternal long-axis view to obtain the flow tracings [Figure 4A]. The peak pulmonary artery velocity (PAV) was unaltered post mTAC surgery (710.5 ± 32.1 mm/s in mTAC *vs*. 747.2 ± 38.5 mm/s in sham, *P* = 0.44) [Figure 4B]. Peak pulmonary flow pressure gradient (PAPG) was also unchanged after mTAC (2.05 ± 0.20 mmHg in mTAC *vs*. 2.26 ± 0.23 mmHg in sham, *P* = 0.45) [Figure 4C]. Finally, pulmonary artery velocity time integral (VTI) did not show a significant change after aortic constriction (20.8 ± 2.10 mm in mTAC *vs*. 27.04 ± 1.99 mm in sham, *P* = 0.1) [Figure 4D]. Overall, our data reveal that mTAC surgery does not cause RV remodeling or pulmonary hypertension in mice.

**Figure 5.**
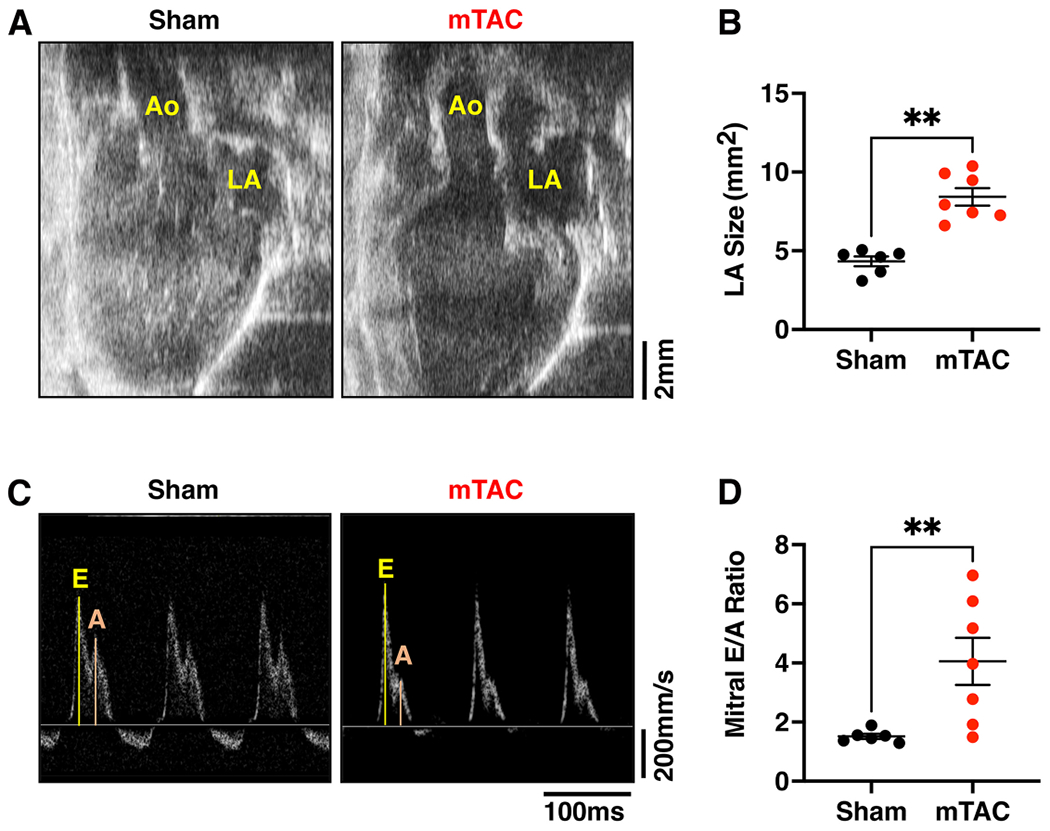
Left atrial remodeling and dysfunction in mTAC model. (A) Representative long-axis echocardiography data of sham (left panel) and mTAC mice (right panel) showing the aorta (Ao), and left atrium (LA). (B) Average value of LA size in sham (black dots, *n* = 6) and mTAC mice (red dots, *n* = 7). (C) Representative Doppler traces of mitral valve blood flow velocity showing early (E) and atrial (A) waves in sham (left panel) and mTAC mice (right panel). (D) Average of mitral early to atrial waves (E/ A) ratio in sham (black dots, *n* = 6) and mTAC (red dots, *n* = 7). Data shown mean ± SEM. **P < 0.01 using student’s *t*-test.

Next, atrial function and structure were assessed using echocardiographic studies. Representative long-axis echocardiography revealed clear images of the aortic root (Ao) and left atrium (LA) at 8 weeks post-surgery in mTAC and sham mice [Figure 5A]. The left atrial (LA) size was significantly larger in mTAC mice compared to sham mice [Figure 5B, *P* < 0.01]. No changes were observed in the right atria (RA) size in mTAC mice (not shown). The atrial contractile function was studied using echo-Doppler in mTAC mice 8 weeks after surgery [Figure 5C]. Representative Doppler images of the mitral valve’s early and late flow peaks revealed an increased mitral early to after waves (E/A) ratio in mTAC *versus* sham mice [Figure 5D, *P* < 0.01], indicating abnormal ventricular filling probably because of decreased atrial contractility.

**Figure 6.**
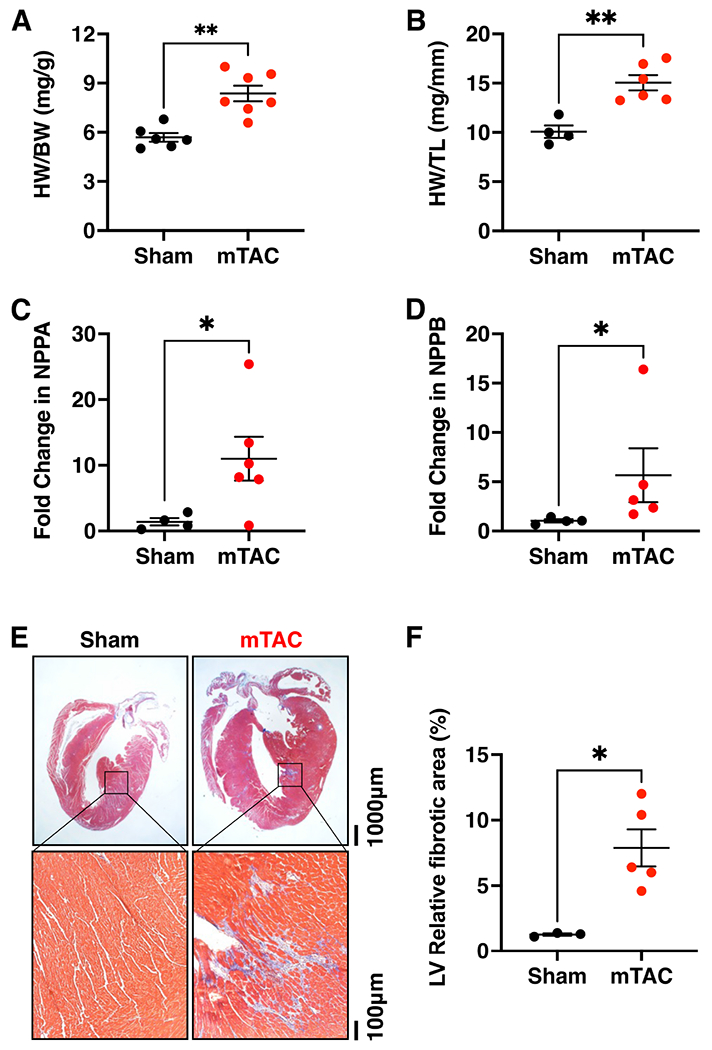
Left ventricular remodeling induced by mTAC. (A and B) Quantification of heart weight to body weight (HW/BW) (A) and heart weight to tibia length ratios (HW/TL) (B) in mTAC *versus* sham mice. Quantification of NPPA (C) and NPPB (D) mRNA expression in mTAC *vs*. sham hearts. (E) Masson trichrome staining of fibrosis in the whole heart (upper panels) and left ventricular histological sections (bottom panels) in sham (left panels) and mTAC mice (right panels). (F) Quantification of left ventricular fibrosis in sham and mTAC mice. Data shown mean ± SEM. *P < 0.05 and **P < 0.01 using student’s *t*-test.

Finally, cardiac structure was analyzed in mTAC and sham mice 8 weeks after surgery. There were no changes in body weight (BW) comparing both groups of mice [Table 2]. Heart weight (HW) was found to be significantly higher in mTAC mice than in sham mice [Table 2, *P* < 0.001]. Thus, mTAC mice had significant cardiac hypertrophy as indicated by a higher HW-to-BW ratio [HW/BW, Figure 6A, *P* < 0.01] and HW to tibia length (TL) ratio [HW/TL, Figure 6B, *P* < 0.01] compared to sham mice. The mTAC hearts showed higher expression levels of NPPA and NPPB compared to sham hearts [Figure 6C and D, *P* < 0.05]. Furthermore, lung weight (LW) was significantly greater in mTAC mice *versus* sham [Table 2, *P* < 0.01]. While there was a trend towards an increased LW-to-BW (LW/BW) ratio, this did not reach a statistically significant difference between both groups [Table 2]. However, the LW-to-TL (LW/TL) ratio was significantly higher in mTAC mice than in sham mice [Table 2, *P* < 0.001], consistent with pulmonary edema. To determine the contribution of fibrosis to the heart hypertrophy, histological sections from 3 sham and 5 mTAC mouse hearts were analyzed. Ventricular fibrosis was visualized by staining longitudinal cardiac sections using Masson’s trichrome [Figure 6E]. The relative area occupied by fibrosis in the left ventricle (LV) was significantly higher in hearts from mTAC mice *versus* sham mice [Figure 6F, *P* < 0.05]. However, no significant differences in atrial fibrosis were found when comparing both groups (data not shown).

## DISCUSSION

In the present study, we characterized ventricular and atrial remodeling and functional alterations in an improved, minimally invasive transverse aortic constriction (mTAC) mouse model of pressure overload-induced heart failure. Key findings are (i) that intubation of the animal guarantees a high intraoperative survival rate in the mTAC model; (ii) aortic constriction increases right carotid blood flow and decreases left carotid blood flow; (iii) HF develops starting from two weeks after surgery; and (iv) mTAC induces atrial remodeling and dysfunction 8 weeks after surgery. Thus, here we show mTAC is a highly reproducible surgical model to study HF and its progression. Given that these mice also develop atrial remodeling, it would be interesting to determine whether this model could also be used to study the development of AF secondary to HF.

The most used surgical procedure to induce pressure overload is conventional TAC (cTAC), which is a very invasive procedure that includes thoracotomy^[16,17]^. Furthermore, cTAC has been reported to have a higher intraoperative mortality rate^[8]^. Thus, the mTAC procedure was developed as a less invasive model of ventricular pressure overload^[10,11,18]^. The perioperative mortality associated with the mTAC model was previously reported to be around 10%^[10]^, similar to the one observed for our current procedure. This lower procedure-related mortality rate is probably related to reducing pulmonary injury, lung collapse, and inflammation. While intubation could potentially help mitigate the detrimental effects of lung collapse in the cTAC model, overall mortality still exceeds the one observed with the mTAC model. Our mTAC model was associated with a 70% postoperative survival rate, which is comparable to prior mTAC studies^[10,19]^. Mortality that occurred between 2 and 56 days after surgery was most likely caused by infection or complications of HF, including arrhythmogenic cardiac death and pump failure.

The present mTAC model allows for constriction of the aorta without opening the chest cavity. Such a minimally invasive, closed-chest surgical procedure makes the model more similar to the actual pathology seen in human patients^[20]^, which improves the relevance of the mechanistic observations made in this model. As we previously demonstrated in a recent paper, the opening of the chest cavity *per se* induces major changes in heart function, leading to the development of arrhythmias due to the inflammatory response induced by surgery^[3]^. Using a closed-chest model, we minimized the activation of inflammation processes around the heart, which have been demonstrated to affect cardiac function in prior work^[21]^.

The effectiveness of the model was assessed one week after surgery by the measurement of the RC/LC flow ratio. Our model showed a RC/LC flow ratio of 5.9, corresponding to a moderate-to-severe pressure gradient, similar to what were previously reported for the cTAC model^[8]^. This result supports the effectiveness of the ligation in the closed-chest TAC method. Furthermore, we found cardiac hypertrophy and fibrosis in mTAC mice when collecting the hearts at 8 weeks post-surgery consistent with a HF phenotype.

It is well-known that pressure overload-TAC models are characterized by cardiac remodeling and dysfunction^[22]^. However, it has also been shown that the phenotype observed following cTAC in wildtype C57BL/6J mice can be highly variable, with almost 70% of animals not developing HF in one study^[23]^. The lack of consistency in the progression to HF could be due to aortic stenosis ranging from −0.3 to −0.6 mm^[24,25]^. Some authors support the use of O-rings for a more consistent constriction^[7,26]^, but it is essential to consider that the constriction depends not only on the diameter of the ring used but also on the original diameter of the aortic arch which differs between animals. The present study did not evaluate the stenosis reached with this new procedure, but the better access to the aortic arch with this method might explain a greater consistency of the ligation. We strongly recommend a Doppler study around 1-week post-surgery to obtain a quantitative assessment of the degree of functional stenosis, which might be a better predictor of subsequent remodeling^[27]^.

We observed that mTAC mice exhibited a progressive decrease in EF starting during the second week after surgery, like in previous studies^[18,28]^. In the present study, all mTAC animals that reached the endpoint developed HF, with an EF of around 30% at 8 weeks after surgery [Figure 3C]. These findings demonstrated that mTAC is an excellent model for studying the physiopathology of HF and is a consistent surgical procedure to induce HF progression.

The echocardiography techniques utilized for the study also revealed for the first time left atrial remodeling and contractile abnormalities associated with mTAC. Left atrial remodeling and contractile dysfunction may occur due to alterations in left ventricular function. In this sense, systolic^[29,30]^ and diastolic^[31,32]^ HF increase left atrial pressure. It has been recently shown that left atrial size is a good predictor of HF in human patients^[33]^. Atrial hypertrophy^[9,28,34]^ and increased left atrial fibrosis^[35,36]^ have been previously described in the cTAC model, but no analysis of atria structure or function has been reported so far in the mTAC model. Several studies have focused on ventricular alterations, while atrial pathology is often disregarded. Here we showed that the present pressure overload model not only induces left atrial enlargement but also left atrial contractile dysfunction. Thus, the mTAC model might be a good and reproducible model of atrial cardiomyopathy, a condition commonly observed in patients with HF^[37]^. Interestingly, atrial size has a significant predictive value for incident cardiovascular disease and mortality^[38]^. This association is not limited to patients with established cardiovascular disease but also extends to other pathologies such as cancer^[39]^ or diabetes^[40]^. However, the comparison of cTAC and mTAC in the same study would demonstrate the advantages of using mTAC, which is a limitation of the present study.

In conclusion, we developed and characterized an improved, minimally invasive TAC surgical model of cardiac pressure overload that was characterized by low intraoperative mortality, consistent progressive cardiac remodeling and left ventricular contractile dysfunction, as well as left atrial cardiomyopathy. This mTAC model caused the development of HF with a reduced ejection fraction starting from two weeks post-surgery in 100% of mice. Moreover, this mTAC model developed hemodynamically significant atrial cardiomyopathy 8 weeks after the surgery. All these findings support the use of this improved mTAC model for a mechanistic understanding of HF and HF-associated AF and the development of new therapeutic drugs treating these conditions.

## Figures and Tables

**Table 1.** Echocardiography parameters in mTAC *versus* sham mice

	Sham (*n* = 6)	mTAC (*n* = 7)	*P*-value
**Baseline**			
Heart rate (bpm)	472.7 ± 16.3	457.2 ± 12.0	0.447
ESV (μL)	27.8 ± 1.28	30.0 ± 1.95	0.397
EDV (μL)	82.7 ± 2.83	84.0 ± 3.64	0.794
SV (μL)	54.9 ± 1.91	54.0 ± 2.03	0.754
CO (mL/min)	25.9 ± 1.14	24.7 ± 1.21	0.488
LV mass (mg)	106.2 ± 10.0	101.1 ± 8.81	0.710
LVAW;s (mm)	1.18 ± 0.07	1.05 ± 0.05	0.123
LVAW;d (mm)	0.63 ± 0.04	0.58 ± 0.03	0.353
**2-weeks**			
Heart rate (bpm)	481.5 ± 19.9	505.3 ± 17.1	0.382
ESV (μL)	29.5 ± 3.21	39.4 ± 2.77	0.037*
EDV (μL)	81.0 ± 7.95	77.9 ± 4.28	0.717
SV (μL)	51.6 ± 4.9	38.5 ± 2.09	0.019*
CO (mL/min)	24.5 ± 1.82	19.3 ± 0.87	0.015*
LV mass (mg)	84.3 ± 7.80	123.6 ± 12.0	0.026*
LVAW;s (mm)	0.94 ± 0.04	1.07 ± 0.06	0.136
LVAW;d (mm)	0.52 ± 0.04	0.72 ± 0.04	0.004*
**4-weeks**			
Heart rate (bpm)	494.7 ± 19.8	533.7 ± 17.0	0.160
ESV (μL)	30.9 ± 2.58	55.8 ± 4.36	0.001*
EDV (μL)	82.9 ± 4.39	94.5 ± 5.18	0.130
SV (μL)	52.0 ± 1.91	38.6 ± 2.05	0.001*
CO (mL/min)	25.6 ± 1	20.5 ± 1.02	0.004*
LV mass (mg)	85.8 ± 8.79	127.8 ± 9.96	0.010*
LVAW;s (mm)	0.95 ± 0.05	0.96 ± 0.11	0.946
LVAW;d (mm)	0.53 ± 0.02	0.68 ± 0.08	0.118
**6-weeks**			
Heart rate (bpm)	466.8 ± 17.6	542.6 ± 14.7	0.007*
ESV (μL)	31.7 ± 2.82	61.5 ± 4.48	< 0.001*
EDV (μL)	80.4 ± 6.69	97.2 ± 3.16	0.036*
SV (μL)	48.7 ± 4.17	35.7 ± 2.01	0.013*
CO (mL/min)	22.6 ± 1.72	19.3 ± 1.14	0.132
LV mass (mg)	95.9 ± 6.29	104.6 ± 5.05	0.301
LVAW;s (mm)	1.00 ± 0.04	0.84 ± 0.04	0.012*
LVAW;d (mm)	0.62 ± 0.02	0.64 ± 0.03	0.456
**8-weeks**			
Heart rate (bpm)	522.5 ± 18.2	552.2 ± 10.1	0.166
ESV (μL)	29.9 ± 0.91	74.7 ± 4.64	< 0.001*
EDV (μL)	78.3 ± 2.53	110.6 ± 6.44	0.001*
SV (μL)	48.5 ± 1.74	35.9 ± 2.53	0.002*
CO (mL/min)	25.3 ± 1.15	19.9 ± 1.58	0.022*
LV mass (mg)	95.7 ± 4.73	98.7 ± 8.57	0.772
LVAW;s (mm)	0.95 ± 0.06	0.67 ± 0.06	0.005*
LVAW;d (mm)	0.57 ± 0.02	0.53 ± 0.03	0.387

Bpm, beats-per-minute; ESV: End-systolic volume; EDV: end diastolic volume; SV: stroke volume; CO: cardiac output; LV: left ventricular; LVAW;s: left ventricular anterior wall thickness in systole; LVAW;d: left ventricular anterior wall thickness in Asterisks mark statistically significant values according to the student’s *t*-test.

**Table 2.** Macroscopic parameters in mTAC *versus* sham mice at 8-week post-surgery

	Sham (*n* = 6)	mTAC (*n* = 7)	*P*-value
Body weight (BW, g)	32.4 ± 1.1	32.4 ± 0.8	0.601
Heart weight (HW, mg)	184.3 ± 11.3	269.7 ± 11.9	0.001*
Tibia length (TL, mm)	18.3 ± 0.3	17.9 ± 0.1	0.115
Lung weight (LW, mg)	153.2 ± 3.4	173.7 ± 7.7	0.004*
LW/BW ratio	4.7 ± 0.2	5.4 ± 0.2	0.073
LW/TL ratio	8.4 ± 0.2	9.7 ± 0.4	0.001*

Asterisks mark statistically significant values according to the student’s *t*-test.

## Data Availability

Upon submission, authors agree to make any materials, data, code, and associated protocols available upon request.
